# Disseminated Septic Emboli From Aortic Valve Infective Endocarditis in an Immunocompromised Patient: A Case Report

**DOI:** 10.7759/cureus.113567

**Published:** 2026-07-29

**Authors:** Mandeep Singh, Sharmila Seeralan, Mayuran Seeralan, Olanrewaju Oladipo

**Affiliations:** 1 General Internal Medicine, Princess Alexandra Hospital, Harlow, GBR; 2 Geriatrics, Princess Alexandra Hospital NHS Trust, Harlow, GBR

**Keywords:** cancer immunotherapy, cerebral abscess, immunosuppression, infective endocarditis, septic emboli

## Abstract

Disseminated sepsis with septic embolic disease can present with a wide spectrum of clinical manifestations and may closely mimic metastatic malignancy or immune-related adverse events in oncology patients. We report the case of a 50-year-old woman with cervical squamous cell carcinoma receiving pembrolizumab and infliximab who presented with fever, progressive blistering skin lesions, non-pruritic rash, and new right-sided neurological deficits. Initial concerns included toxic epidermal necrolysis, immunotherapy-related toxicity, metastatic disease, and severe infection.

Examination revealed haemorrhagic blisters involving the fingers and toes, truncal rash, and transient right-sided weakness. Blood investigations demonstrated markedly elevated inflammatory markers. Imaging showed a cavitating left lower lobe lung lesion with air-fluid level, bilateral pulmonary infiltrates, hepatic lesions, and ring-enhancing lesions within the left frontal and parietal lobes concerning for cerebral abscesses or metastases. MRI brain findings favoured cerebral abscesses with surrounding vasogenic oedema. Wound cultures from hand and foot lesions grew *Streptococcus pyogenes* (Group A *Streptococcus*). Multidisciplinary input from dermatology, oncology, respiratory medicine, microbiology, vascular surgery, gastroenterology, and neurosurgery guided management.

The patient initially received empirical piperacillin-tazobactam and gentamicin for severe sepsis. Following multidisciplinary microbiology review, antimicrobial therapy was escalated to meropenem and teicoplanin to provide broad-spectrum coverage while the diagnostic evaluation was ongoing because of extensive cerebral, pulmonary, and soft tissue infection in an immunocompromised host. The patient demonstrated significant clinical and biochemical improvement, and pembrolizumab and infliximab were withheld. Neurosurgical intervention was not considered appropriate due to the extent of systemic disease and favourable response to medical management.

This case highlights the diagnostic complexity of disseminated infection in immunocompromised oncology patients, particularly when septic emboli and cerebral abscesses mimic metastatic disease or immune-related adverse events. Early multidisciplinary assessment and prompt antimicrobial therapy were essential in achieving clinical stabilisation.

## Introduction

Infective endocarditis (IE) remains a serious condition associated with substantial morbidity and mortality despite advances in antimicrobial therapy and cardiac imaging. Inpatient mortality rates vary between 13% and 25%, and in recent studies, it was found that 9-20% of patients died a year post-discharge [1]. Embolic complications occur in approximately 20-40% of patients and represent one of the major causes of adverse outcomes [2]. Septic emboli may involve multiple organs, including the brain, lungs, spleen, liver, kidneys, and skin, often producing a complex clinical picture that can delay diagnosis.

Immune checkpoint inhibitors such as pembrolizumab have significantly improved progression-free and overall survival in patients with persistent, recurrent, or metastatic cervical cancer, as demonstrated in the KEYNOTE-826 trial [3]. However, their use is associated with a broad range of immune-related adverse events affecting the skin, gastrointestinal tract, endocrine organs, lungs, and nervous system. Cutaneous manifestations are among the most common immune-related adverse events and can range from mild maculopapular eruptions to severe cutaneous adverse reactions such as Stevens-Johnson syndrome (SJS) and toxic epidermal necrolysis (TEN). Although SJS/TEN is rare, it is potentially life-threatening and should be considered in patients receiving immune checkpoint inhibitors who develop blistering skin lesions or mucosal involvement [4,5]. In patients receiving immunotherapy, new skin lesions accompanied by systemic symptoms often raise immediate concern for treatment-related toxicity.

In this patient, the combined effects of underlying malignancy, previous chemotherapy and chemoradiotherapy, infliximab therapy, and recent corticosteroid exposure were considered clinically relevant contributors to immunosuppression and susceptibility to severe infection. Disseminated sepsis with septic embolisation may involve multiple organs, including the lungs, liver, skin, and central nervous system, and can closely resemble metastatic disease both clinically and radiologically. IE may therefore present with rapidly progressive multisystem disease, making early recognition and multimodality imaging essential for timely diagnosis and management [1].

Brain abscesses remain uncommon but potentially life-threatening complications of systemic infection [6]. Their radiological appearance, especially ring-enhancing lesions with surrounding oedema, can overlap significantly with cerebral metastases. In patients with known malignancy, distinguishing between infective and metastatic lesions is critical because management strategies differ substantially [6].

We present a diagnostically challenging case of disseminated sepsis with septic embolic disease and cerebral abscesses in a patient with metastatic cervical squamous cell carcinoma receiving pembrolizumab and infliximab. The patient initially presented with blistering skin lesions and a truncal rash suggestive of possible immunotherapy-related toxicity or TEN. Subsequent investigations demonstrated extensive infective pathology, including a pulmonary abscess, septic embolic complications and cerebral abscesses, with wound cultures supporting probable invasive Group A *Streptococcus* infection. This case highlights the importance of maintaining a broad differential diagnosis and adopting a multidisciplinary approach in complex oncology patients.

## Case presentation

A 50-year-old woman with a background of cervical squamous cell carcinoma presented to the emergency department with progressive blistering skin lesions, rash, fever, and neurological symptoms. She had previously undergone neoadjuvant chemotherapy followed by chemoradiotherapy with brachytherapy and was receiving pembrolizumab every six weeks for stable disease. Her medical history also included ulcerative colitis treated with infliximab, asthma, and hypothyroidism.

On examination, the patient appeared systemically unwell with a marked inflammatory response. Resolving haemorrhagic blisters were present over the right second finger, left third finger, and right fifth toe, together with a resolving blanching erythematous rash over the trunk. There was no mucosal involvement or epidermal detachment. Neurological examination demonstrated mild expressive dysphasia with right-sided upper and lower limb weakness (Medical Research Council grade 4/5), without facial asymmetry or sensory deficit. Cardiovascular examination revealed normal heart sounds without an audible murmur. Respiratory examination demonstrated mild bibasal crackles with reduced air entry at the left lung base. Initial laboratory investigations showed a marked inflammatory response, including a C-reactive protein of 413 mg/L, white cell count of 18.7 × 10^9^/L, and procalcitonin of 2.5 ng/mL.

The patient's laboratory and microbiological findings are summarised in Table 1, demonstrating a marked systemic inflammatory response, microbiological evidence of infection, and subsequent improvement in inflammatory markers following antimicrobial therapy.

**Table 1:** Laboratory and microbiological findings Laboratory and microbiological findings demonstrated a severe systemic infective response with elevated inflammatory markers, neutrophilic leukocytosis, positive pneumococcal urinary antigen, and wound cultures identifying *S. pyogenes* (+++ indicates heavy or abundant growth). Serial inflammatory markers showed significant improvement following antimicrobial therapy.

Investigation	Findings	Interpretation	Reference range
C-reactive protein (CRP)	413 → progressively reduced to 15 mg/L	Marked inflammatory response with improvement following treatment	<5 mg/L
Procalcitonin	2.5	Supportive of severe bacterial infection	<0.1 ng/mL
White cell count (WCC)	18.7 ×10^9^/L with neutrophilia	Acute infective/inflammatory response	4-11 ×10^9^/L
Blood cultures	No growth	Obtained after commencement of empirical antibiotics	-
Pneumococcal urinary antigen	Positive	Evidence of pneumococcal infection	-
Wound swab culture	*Streptococcus pyogenes* (+++) Coagulase-negative staphylococci	Significant soft tissue infection with Group A *Streptococcus* growth	-

**Figure 1:**
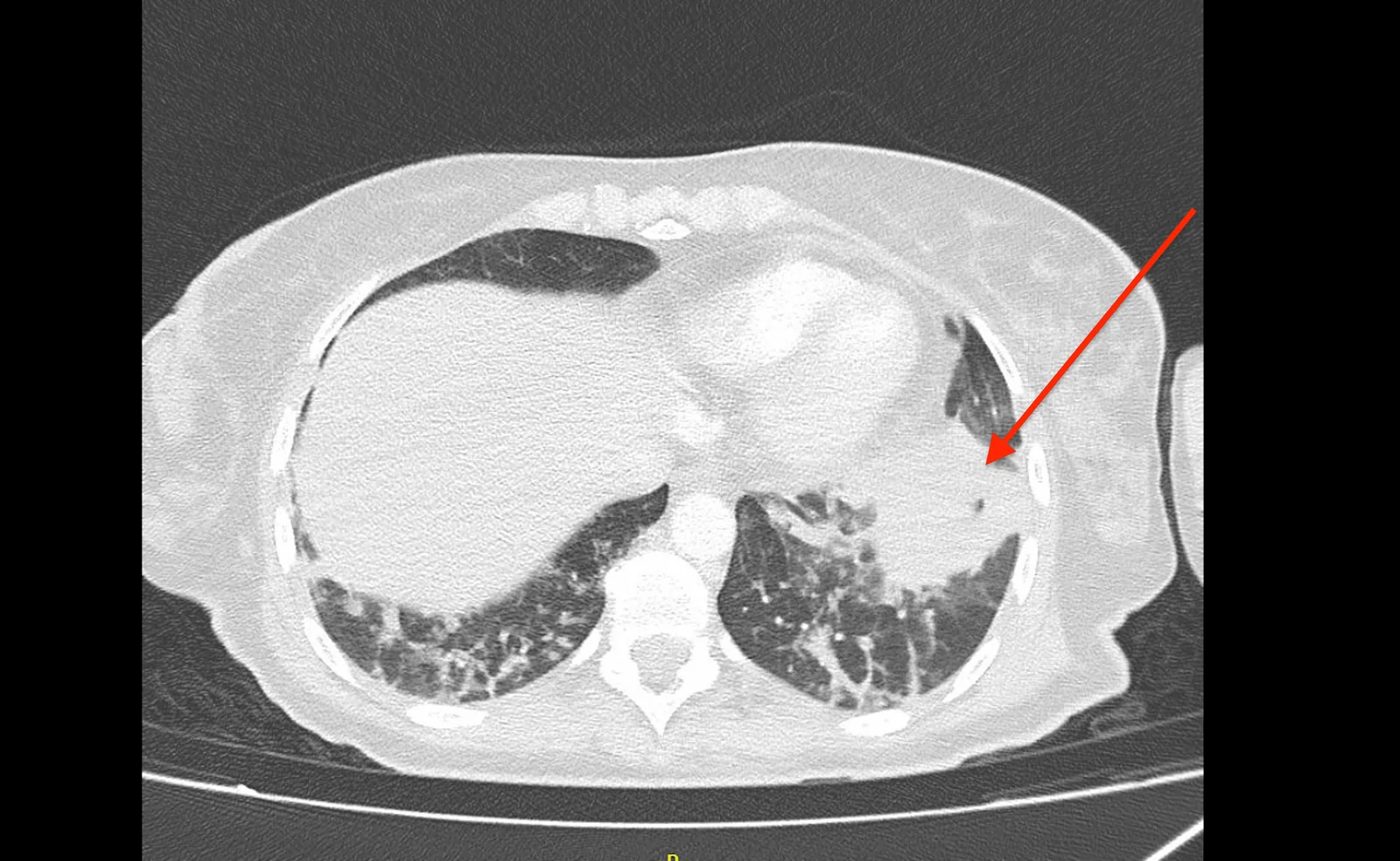
Contrast-enhanced computed tomography (CT) thorax demonstrating septic pulmonary embolisation Axial CT image of the thorax showing peripheral nodular air-space consolidation within the left lower lobe (arrow), consistent with septic pulmonary embolisation. Additional bilateral patchy bronchopneumonic infiltrates were also present.

**Figure 2:**
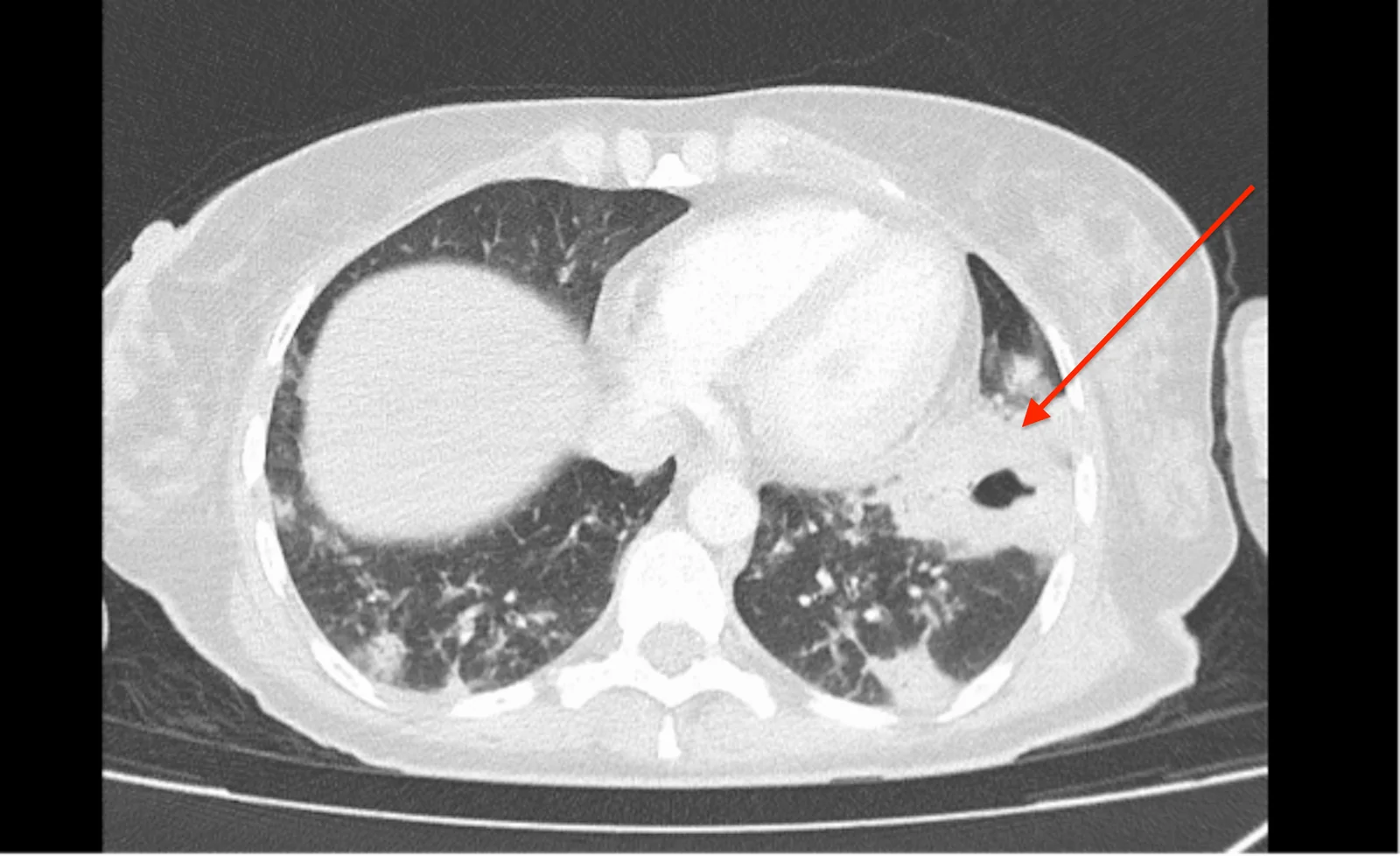
Contrast-enhanced computed tomography (CT) thorax demonstrating cavitating lung abscess Axial CT image demonstrating a thick-walled cavitating lesion containing an air-fluid level within the left lower lobe (arrow), consistent with a lung abscess secondary to septic embolisation. Surrounding consolidation and bilateral bronchopneumonic changes were also evident.

**Figure 3:**
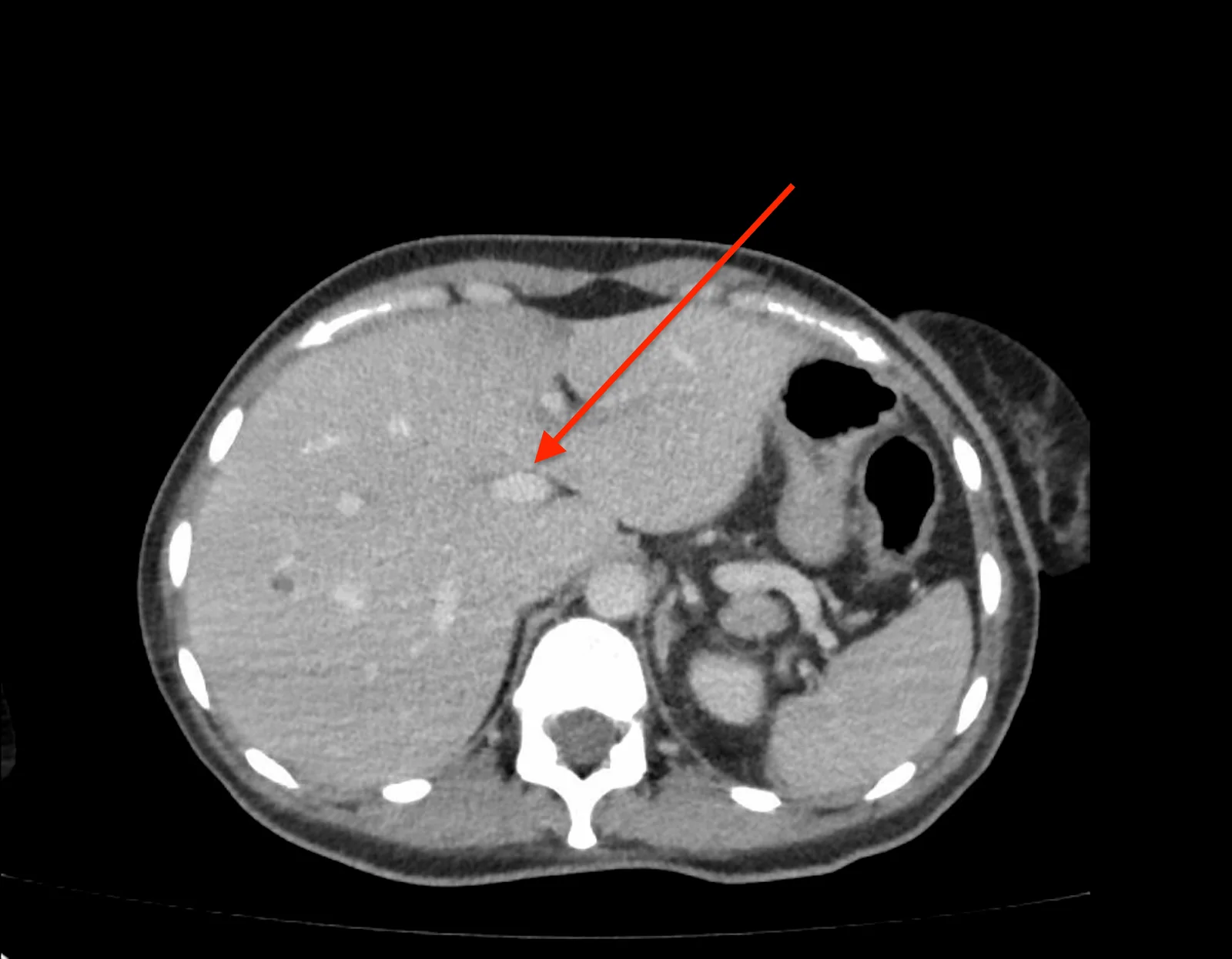
Contrast-enhanced computed tomography (CT) abdomen demonstrating hepatic septic embolus Contrast-enhanced CT abdomen demonstrating a hypodense lesion within the right hepatic lobe (arrow), suspicious for hepatic septic embolus (or hepatic abscess) in the setting of disseminated infection. Additional small hypodense lesions were seen in both hepatic lobes.

**Figure 4:**
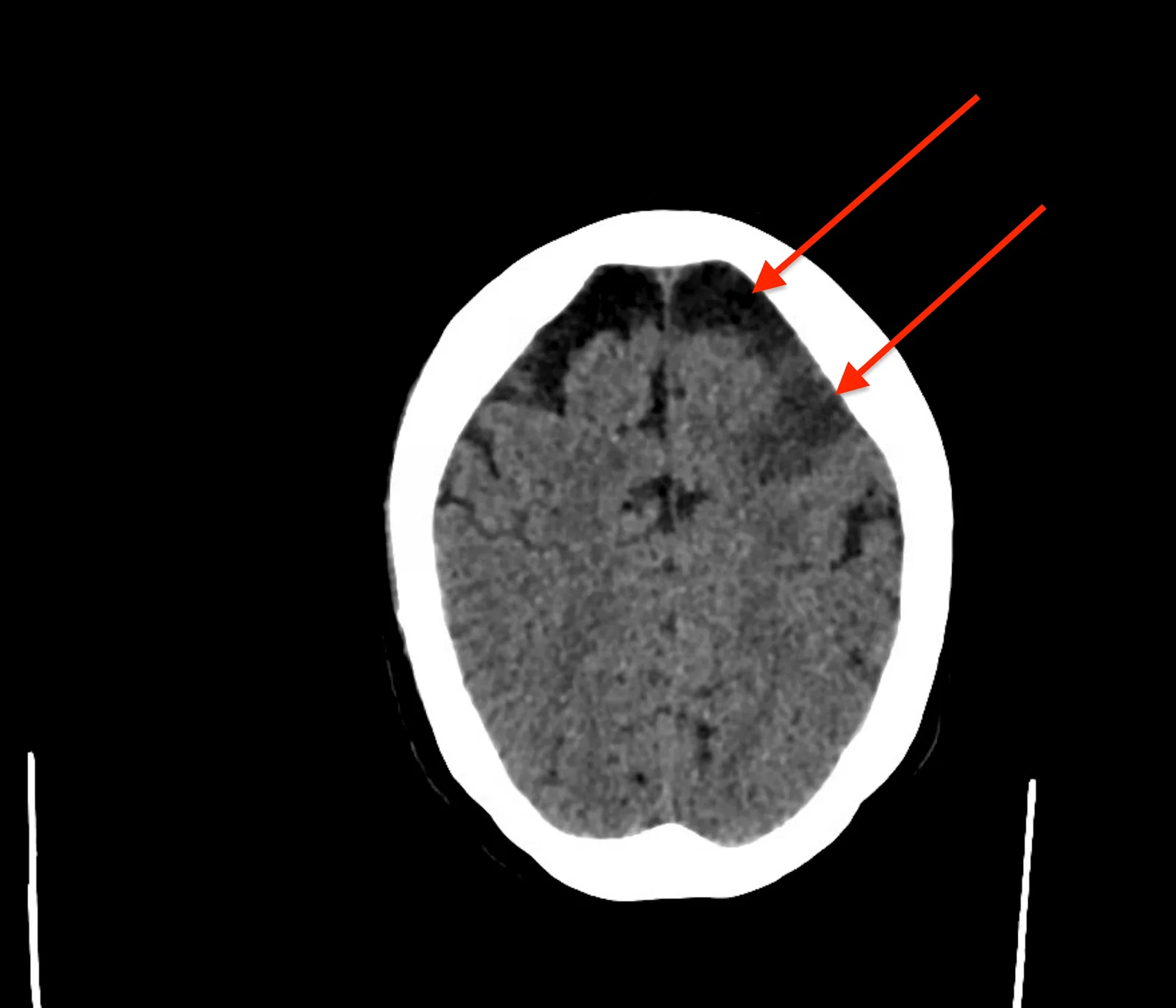
Non-contrast computed tomography (CT) head demonstrating septic embolic cerebral lesions Axial non-contrast CT image demonstrating wedge-shaped cortical and subcortical hypodensity involving the left frontal lobe (upper red arrow) with mild adjacent mass effect (lower red arrow), raising suspicion for septic embolic infarction. Subsequent MRI confirmed ring-enhancing cerebral abscesses.

**Figure 5:**
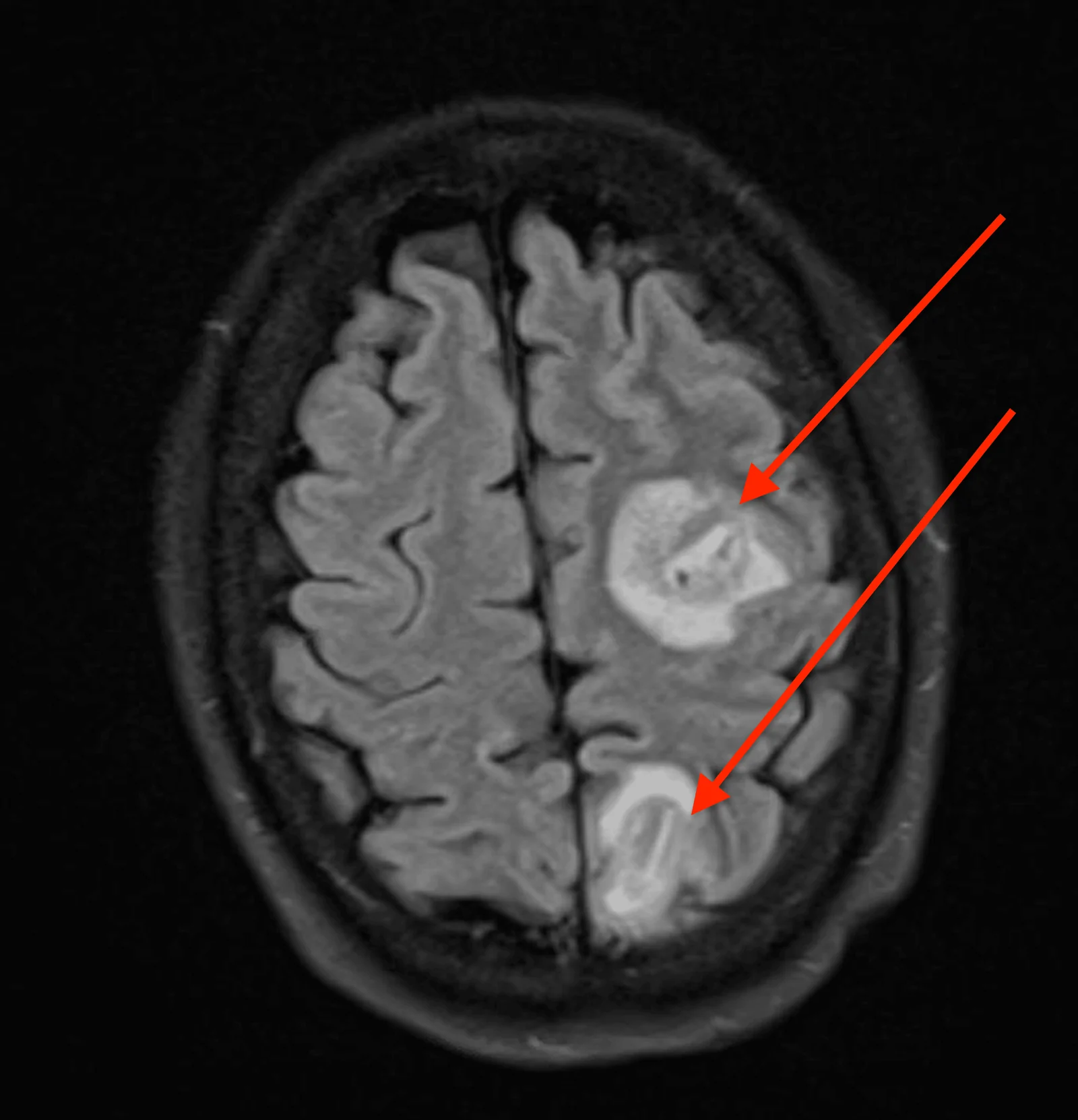
Magnetic resonance imaging (MRI) of the brain demonstrating cerebral abscesses Post-contrast axial MRI demonstrates two ring-enhancing lesions involving the left frontal (upper red arrow) and left parietal lobes (lower red arrow) with surrounding vasogenic oedema and diffusion restriction, findings favouring cerebral abscesses over metastatic disease in the clinical context of disseminated infection.

Computed tomography (CT) of the chest, abdomen, and pelvis demonstrated bilateral pulmonary infiltrates (Figure 1), a thick-walled cavitating lesion within the left lower lobe with surrounding consolidation (Figure 2), and multiple hepatic lesions suspicious for septic emboli or metastatic disease (Figure 3). Additional findings included colitis and soft tissue inflammatory changes in the right thigh. CT head imaging revealed two ring-enhancing lesions in the left frontal and parietal lobes associated with vasogenic oedema (Figure 4). MRI findings were more suggestive of cerebral abscesses, although metastases remained in the differential diagnosis (Figure 5).

The radiological and cardiac imaging findings are summarised in Table 2, highlighting disseminated infection with pulmonary, cerebral, and cardiac involvement, including findings consistent with aortic valve IE and multiple septic embolic complications.

**Table 2:** Radiological and cardiac imaging findings Radiological and cardiac imaging findings demonstrating disseminated infection with multi-organ involvement, including pulmonary and cerebral septic complications. Advanced imaging identified lung abscess, cerebral abscesses, and embolic infarcts, while transoesophageal echocardiography confirmed aortic valve infective endocarditis. LV: left ventricular; EF: ejection fraction

Investigation	Key Findings	Impression
Chest X-ray	Left lower zone airspace shadowing	Consistent with infective consolidation
CT head	• Wedge-shaped cortical and subcortical hypodensities in left frontal lobe • Small hyperdense focus suggestive of microhaemorrhage • Mild mass effect without midline shift	Features suggestive of embolic cerebral infarcts
CT thorax, abdomen and pelvis	• Left lower lobe cavitating lesion (4.3 × 4.7 cm) with air-fluid level • Bilateral bronchopneumonia • Liver hypodense lesion (4 × 1.7 cm) • Left-sided colitis • Right thigh soft tissue oedema	Disseminated infective process with lung abscess, probable liver abscess and septic embolisation
MRI brain	• Two 19 mm ring-enhancing lesions in left frontal and parietal lobes • Diffusion restriction and vasogenic oedema • Additional small occipital lesion	Consistent with cerebral abscesses (metastatic disease less likely)
Follow-up MRI brain (two months after)	Persistent but stable lesions • No new lesions	Stable appearances with no disease progression
Initial transthoracic echocardiogram (TTE)	Normal LV systolic function (EF 55-60%) • No vegetations visualised	No initial evidence of valvular vegetation
Transoesophageal echocardiogram (TOE)	Vegetation identified on non-coronary cusp of aortic valve • Mild aortic regurgitation	Confirmed aortic valve infective endocarditis
Follow-up echocardiogram	No visible vegetation • Reduced LV systolic function (EF 40-45%)	Resolution of vegetation with residual LV dysfunction

Dermatology initially considered the cutaneous lesions to represent resolving TEN secondary to a drug reaction. However, following multidisciplinary review, the oncology and dermatology teams concluded that the overall presentation was more consistent with disseminated infection than pembrolizumab-related toxicity.

Peripheral blood cultures were obtained following commencement of empirical intravenous piperacillin-tazobactam and gentamicin but remained negative throughout the admission. Wound swabs obtained from the haemorrhagic finger and toe lesions subsequently grew *Streptococcus pyogenes* (Lancefield Group A), together with scanty coagulase-negative staphylococci, providing microbiological evidence of invasive infection.

**Figure 6:**
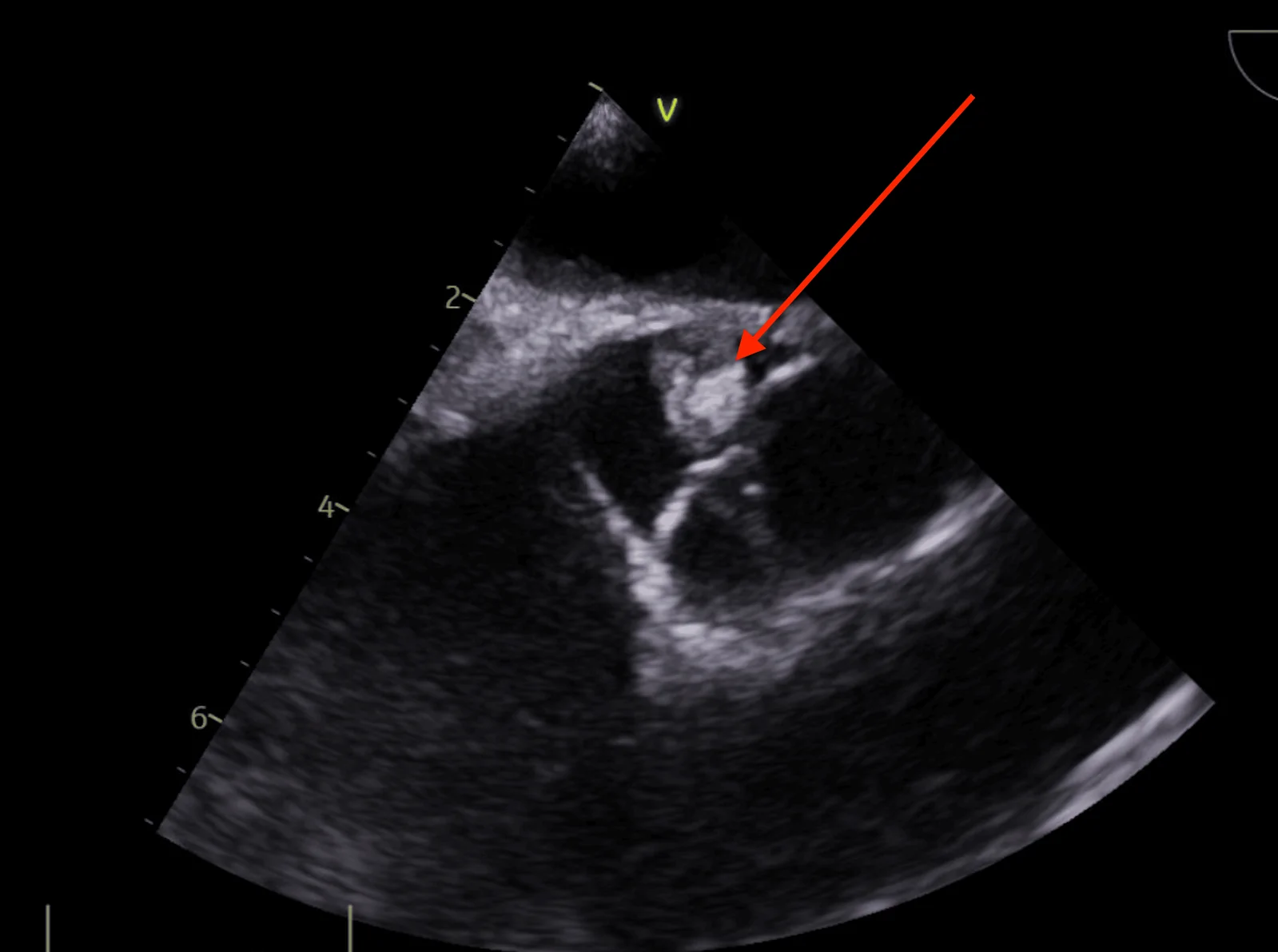
Transoesophageal echocardiography demonstrating native aortic valve infective endocarditis Mid-oesophageal views showing a mobile vegetation attached to the non-coronary cusp of the trileaflet aortic valve (arrow), with associated mild aortic regurgitation, confirming the diagnosis of aortic valve infective endocarditis.

The patient underwent extensive multidisciplinary evaluation involving oncology, microbiology, respiratory medicine, vascular surgery, gastroenterology, cardiology, dermatology, and neurosurgery. Differential diagnoses included disseminated sepsis with septic emboli, IE, metastatic disease, and immune-related adverse events. Transthoracic echocardiography demonstrated preserved left ventricular systolic function without visible vegetations. Subsequent transoesophageal echocardiography identified a vegetation on the non-coronary cusp of the aortic valve with mild aortic regurgitation, confirming native aortic valve IE (Figure 6).

The patient initially received empirical piperacillin-tazobactam and gentamicin for severe sepsis. Following multidisciplinary review by microbiology, antimicrobial therapy was escalated to high-dose meropenem and teicoplanin to provide broad-spectrum coverage while awaiting diagnostic clarification because of disseminated cerebral, pulmonary, and soft tissue infection in an immunocompromised host.

For confirmed susceptible streptococcal IE, narrower penicillin-based treatment would ordinarily be considered; however, broader empirical therapy was considered appropriate while the extent and microbiology of the disseminated infection remained uncertain. Neurosurgical intervention was not considered appropriate due to the patient’s overall condition and imaging appearances favouring infective lesions amenable to medical management. Following prolonged intravenous antimicrobial therapy, the patient's neurological deficits, inflammatory markers, and radiological findings improved significantly. Follow-up transthoracic echocardiography demonstrated complete resolution of the aortic valve vegetation with no evidence of progressive valvular regurgitation or structural valve destruction. However, left ventricular systolic function had declined, with an estimated ejection fraction of 40-45%. In the absence of ongoing valvular dysfunction, this was considered most consistent with sepsis-associated cardiomyopathy, although transient inflammatory myocardial dysfunction remained a differential diagnosis. Following cardiology review, conservative medical management was recommended, as there was no indication for surgical intervention given resolution of the vegetation and the absence of severe valvular dysfunction or structural destruction.

The patient completed prolonged intravenous antimicrobial therapy, remained clinically stable, and was discharged with outpatient follow-up involving cardiology, infectious diseases, oncology, and repeat imaging.

## Discussion

This case highlights the diagnostic complexity of disseminated septic embolic disease in an immunocompromised oncology patient, where infection, metastatic progression, and immune-related toxicity had overlapping clinical and radiological features. The initial blistering lesions and truncal rash raised concern for pembrolizumab-related severe cutaneous toxicity, including SJS/TEN, which has been reported in patients receiving immune checkpoint inhibitors, including cervical cancer patients [5]. Pierre et al. described pembrolizumab-induced SJS/TEN in metastatic cervical squamous cell carcinoma, reinforcing why this was an important early differential in our patient [5].

However, the subsequent finding of aortic valve vegetation on transoesophageal echocardiography, together with lung abscess, liver lesion, cerebral abscesses, and cutaneous lesions, supported the diagnosis of disseminated IE with septic embolisation [1,7].

Although pulmonary septic emboli are classically associated with right-sided IE, pulmonary abscesses may also occur in patients with left-sided disease through secondary haematogenous dissemination, aspiration during severe systemic illness, or concomitant pulmonary infection, particularly in profoundly immunocompromised individuals. In the present case, the cavitating pulmonary lesion was considered part of a disseminated invasive infective process rather than a consequence of direct septic embolisation from the aortic valve alone. Similar multisystem septic embolic presentations have been described recently. Amri et al. reported IE complicated by multiple septic emboli involving the brain, spleen, and spinal cord, emphasising that embolic complications can dominate the clinical picture and require multidisciplinary management [7].

The association of aortic valve endocarditis with both lung and brain abscesses has also been described by Gavaruzzi et al., who reported a patient with a *Streptococcus intermedius* brain abscess, a lung abscess, and native aortic valve endocarditis [8]. Their case is particularly relevant because it demonstrates how simultaneous pulmonary and intracranial abscesses may arise from haematogenous spread and may improve with prolonged antimicrobial therapy, with or without neurosurgical intervention [8].

In our patient, the cerebral ring-enhancing lesions created a major diagnostic dilemma because of her known malignancy. In oncology patients, such lesions are often presumed to represent metastases; however, restricted diffusion on MRI, systemic sepsis, and response to antibiotics favoured a diagnosis of cerebral abscesses. Similar diagnostic challenges have been reported in culture-negative IE complicated by cerebral abscesses and in other cases where IE mimicked intracranial malignancy [6,9].

The cutaneous findings were another unusual feature. Haemorrhagic blisters affecting the digits initially suggested drug-related epidermal necrolysis or immune-mediated bullous dermatitis. However, the absence of mucosal involvement, positive wound cultures for *S. pyogenes*, improvement with antibiotics, and concurrent embolic disease favoured infective cutaneous manifestations. Group A *Streptococcus* brain abscess is rare in adults. Still, it has been reported to mimic stroke or tumour at presentation, supporting the need to consider invasive streptococcal infection when neurological deficits occur alongside systemic sepsis [10].

The diagnosis of probable *S. pyogenes* IE was supported by transoesophageal echocardiographic evidence of native aortic valve vegetation together with disseminated septic embolic disease involving the brain, lungs, liver and skin, heavy growth of Group A *Streptococcus* from wound cultures, and a marked clinical response to antimicrobial therapy. Blood cultures remained negative, most likely because microbiological sampling occurred after initiation of empirical intravenous antibiotics, precluding microbiological confirmation of bacteraemia.

This case also demonstrates the limitations of transthoracic echocardiography in complex endocarditis. The initial transthoracic echocardiogram did not identify vegetations, whereas transoesophageal echocardiography later confirmed a vegetation on the non-coronary cusp of the aortic valve. This reinforces the importance of pursuing transoesophageal imaging when clinical suspicion remains high despite a negative initial study.

According to the 2023 Duke-International Society for Cardiovascular Infectious Diseases (ISCVID) criteria, the patient fulfilled one major imaging criterion through echocardiographic demonstration of an aortic valve vegetation, together with minor criteria including fever and vascular phenomena manifested by cerebral and peripheral embolic lesions. Blood cultures remained negative following prior antimicrobial exposure, and Group A *Streptococcus* was isolated from non-sterile cutaneous wound specimens rather than blood or another qualifying sterile site. The clinical presentation was therefore strongly supportive of IE, although strict classification under the Duke-ISCVID clinical criteria should be interpreted cautiously [9].

Overall, this case adds to the literature by illustrating a rare and diagnostically challenging presentation of probable *S. pyogenes* native aortic valve IE with disseminated septic emboli involving the skin, lungs, liver, and brain in an immunocompromised patient receiving pembrolizumab and infliximab. It underlines three key learning points: first, septic emboli can closely mimic metastatic disease; second, immune-related adverse events should not be diagnosed without excluding infection; and third, early multidisciplinary input and prompt broad-spectrum antimicrobial therapy are essential to improving outcomes in complex oncology patients.

## Conclusions

Disseminated IE with septic embolic disease may closely mimic metastatic malignancy and immune-related adverse events in patients receiving immunotherapy. This case of probable *S. pyogenes* native aortic valve IE highlights the importance of early multidisciplinary assessment, careful interpretation of multimodal imaging and prompt antimicrobial treatment in immunocompromised oncology patients. Clinicians should maintain a high index of suspicion for disseminated infection even when the initial presentation suggests drug toxicity or metastatic progression.
